# Factors Affecting the Usage of Wearable Device Technology for Healthcare among Indian Adults: A Cross-Sectional Study

**DOI:** 10.3390/jcm11237019

**Published:** 2022-11-28

**Authors:** Vathsala Patil, Deepak Kumar Singhal, Nithesh Naik, B. M. Zeeshan Hameed, Milap J. Shah, Sufyan Ibrahim, Komal Smriti, Gaurav Chatterjee, Ameya Kale, Anshika Sharma, Rahul Paul, Piotr Chłosta, Bhaskar K. Somani

**Affiliations:** 1 Department of Oral Medicine and Radiology, Manipal College of Dental Sciences, Manipal, Manipal Academy of Higher Education, Manipal 576104, Karnataka, India; 2 Department of Public Health Dentistry, Manipal College of Dental Sciences, Manipal, Manipal Academy of Higher Education, Manipal 576104, Karnataka, India; 3 Department of Mechanical and Industrial Engineering, Manipal Institute of Technology, Manipal Academy of Higher Education, Manipal 576104, Karnataka, India; 4 iTRUE (International Training and Research in Uro-Oncology and Endourology) Group, Manipal 576104, Karnataka, India; 5 Curiouz TechLab Private Limited, BIRAC-BioNEST, Government of Karnataka Bioincubator, Manipal 576104, Karnataka, India; 6 Department of Urology, Father Muller Medical College, Mangalore 575001, Karnataka, India; 7 Robotics and Urooncology, Max Hospital and Max Institute of Cancer Care, New Delhi 110024, India; 8 Department of Neurosurgery, Mayo Clinic, Rochester, MN 55902, USA; 9 Department of Electrical and Electronics Engineering, Manipal Institute of Technology, Manipal Academy of Higher Education, Manipal 576104, Karnataka, India; 10 Kasturba Medical College, Manipal, Manipal Academy of Higher Education, Manipal 576104, Karnataka, India; 11 Department of Psychology, Amity University, Noida 201313, Uttar Pradesh, India; 12 Department of Radiation Oncology, Massachusetts General Hospital, Harvard Medical School, Boston, MA 02115, USA; 13 Center for Biologics Evaluation and Research (CBER), U.S. Food and Drug Administration, Silver Spring, MD 20993, USA; 14 Department of Urology, Jagiellonian University in Krakow, 31-007 Kraków, Poland; 15 Department of Urology, University Hospital Southampton NHS Trust, Southampton SO16 6YD, UK

**Keywords:** wearable healthcare devices, fitness devices, wearable technology, infotainment, mobile health

## Abstract

Background: Wearable device technology has recently been involved in the healthcare industry substantially. India is the world’s third largest market for wearable devices and is projected to expand at a compound annual growth rate of ~26.33%. However, there is a paucity of literature analyzing the factors determining the acceptance of wearable healthcare device technology among low-middle-income countries. Methods: This cross-sectional, web-based survey aims to analyze the perceptions affecting the adoption and usage of wearable devices among the Indian population aged 16 years and above. Results: A total of 495 responses were obtained. In all, 50.3% were aged between 25–50 years and 51.3% belonged to the lower-income group. While 62.2% of the participants reported using wearable devices for managing their health, 29.3% were using them daily. technology and task fitness (TTF) showed a significant positive correlation with connectivity (*r* = 0.716), health care (*r* = 0.780), communication (*r* = 0.637), infotainment (*r* = 0.598), perceived usefulness (PU) (*r* = 0.792), and perceived ease of use (PEOU) (*r* = 0.800). Behavioral intention (BI) to use wearable devices positively correlated with PEOU (*r* = 0.644) and PU (*r* = 0.711). All factors affecting the use of wearable devices studied had higher mean scores among participants who were already using wearable devices. Male respondents had significantly higher mean scores for BI (*p* = 0.034) and PEOU (*p* = 0.009). Respondents older than 25 years of age had higher mean scores for BI (*p* = 0.027) and Infotainment (*p* = 0.032). Conclusions: This study found a significant correlation with the adoption and acceptance of wearable devices for healthcare management in the Indian context.

## 1. Introduction

Wearable devices are instruments that can be worn on the body, typically on or near the skin, and are equipped with sensors capable of detecting various physiological variables. Wearable technology includes devices that can be placed on the limbs, torso, or head such as watches, bracelets, phones, glasses, head-mounted displays, hearing aids, suits, belts, shoes, and patches that can measure various physiological parameters, which include heart rate, rhythm, blood pressure, oxygen saturation, skin temperature, steps traveled, calorie expenditure estimates, blood glucose levels, and UV radiation exposure [1]. This data can be used for physiological-related research studies, detection of aberrant parameters for clinical diagnosis or prognosis to provide biological feedback to the user thereby aiding in monitoring, and even as an educational tool for promoting health and physical fitness. One of the earliest examples of wearable technology, as it pertains to the field of medicine, are portable hearing aids invented in the 19th century [2]. Norman Holter’s discovery of the first wireless electrocardiogram in 1962 ushered in the era of modern medical wearable gadgets [3,4]. The internet enables health-directed wearable devices to stay connected while continuously measuring and recording data. This system is now referred to as “Connected Health” [5].

Newer studies have aimed at early identification and prediction of inflammatory disease, cancer diagnosis, measuring blood alcohol levels, etc. through smartphone screens. Combining deep neural network-machine learning technology with biological age estimation has further enhanced its feasibility and usage [6,7,8,9,10]. In recent years, the world has seen a wave of adoption of wearable devices even among the middle to high-income socio-economic demographics. A recent systematic review and meta-analysis of multiple randomized controlled trials of consumer wearable activity trackers (CWAT) found that they can improve physical activity in sedentary older adults who are overweight/obese or with chronic respiratory diseases and reduce the systolic blood pressure, waist circumference and low-density cholesterol in individuals with type 2 diabetes mellitus and cardiovascular diseases [11]. Wearable devices such as smartwatches have been seen to benefit psychological wellness in individuals with cognitive disorders [12].

India is now the world’s third-largest market for wearable devices. Several studies have found that an increasing number of individuals are purchasing wearable devices to promote fitness and manage their health [13,14]. A recent study determined that consumers in India are motivated by health and autonomy, health self-efficacy, and technological innovativeness to adopt wearable healthcare devices [15,16]. The COVID-19 pandemic encouraged a rapid, massive expansion of remote health management and firmly established telehealth as an accessible, validated model of healthcare. The data on the pandemic’s effect on actual wearable device use in healthcare settings is limited. Studies examining the perception of wearable device technology among adults in India are limited in the literature. Hence, the present study aimed to analyze the perception of Indian Professionals about wearable device technology in terms of its usage in personal health management.

## 2. Materials and Methods

### 2.1. Data Collection and Ethical Considerations

A cross-sectional study was carried out from January 2022 to May 2022 using an online questionnaire, using an anonymized Google form platform, enquiring about participants’ use of wearable devices for healthcare, socio-demographic factors, and factors affecting the use of wearable devices for healthcare. The technology acceptance model (TAM) and technology and task fitness (TTF) models of technology adoption were used for the survey. Data acquisition and analysis were performed after the approval by the Institutional Ethical committee (ethical approval number FMIEC- 94/2021). Informed consent was obtained from all the participants before the study and the data was analyzed by an independent third party.

The questionnaire gathered information regarding demographics, behavioral intention (BI), perceived usefulness (PU), perceived ease of use (PEOU), subjective feelings about technology, task fitness, connectivity, communication, healthcare, infotainment, fashionability, wearability, and subjective norms. The questionnaire (available as Supplementary Materials) was divided into 11 sections, with 3 items each directed at identifying the subject’s feelings concerning wearable devices according to factors described in the TAM model and factors derived from the TTF models, and was distributed to the participants. The response was documented based on a 5-point answer choice based on the Likert scale as follows: (1)—Strongly Disagree/Very Rarely, (2)—Disagree/Rarely, (3)—Undecided/Occasionally, (4)—Agree/Often, and (5)—Strongly Agree/Very Often.

### 2.2. Survey and Participant Characteristics

Respondents consisted majorly of individuals involved in medical science (undergraduate students, postgraduates, consultant physicians) and the engineering field. The questionnaire was surveyed using the Google Forms platform, which focused on the perception and stated usage of wearable devices by the participants. The inclusion criteria for the study included adults >16 years, able to navigate through the online survey platforms, and comfortable with the interpretation of the English language. An information sheet along with informed consent was displayed and documented respectively at the start of the survey. Participation in this survey was voluntary with no incentives provided to the respondents. Survey data collected via Google Forms was stored on the Google Spreadsheet platform on Google Drive, access to which was limited only to members of the research group.

### 2.3. Data Analysis

The responses to the survey were analyzed using the SmartPLS software version 3.0.M3, with PLS path modeling. Descriptive variables of gender, age, qualification, income, and reports of usage of wearable devices for personal healthcare were expressed as categorical variables. Age data were grouped according to less than 18 years old, 18 to 25 years old, 25 to 50 years old, and older than 50 years. Qualification data were grouped according to (1) “10 + 2 schooling”, (2) “graduate”, (3) “post-graduate”, and (4) “diploma” categories. Income data were grouped from a personal annual income of (1) less than 50,000 Rs to 500,000 Rs. (Lower), (2) 500,000 to 2,500,000 Rs. (Middle), (3) 2,500,000 to 5,000,000 Rs. (Upper Middle) and (4) greater than 5,000,000 Rs. (Elite). Wearable device use-frequency data was grouped into (1) once a year, (2) more than once a year, (3) once in a month, (4) once or twice in 3 months, (5) once or twice in a week, and (6) daily. Wearable device usage for healthcare was assessed using a binary “yes” or “no” response. Usage frequency data were grouped according to (1) daily, (2) once or twice in a week, (3) once in a month, (4) once or twice in 3 months, (5) more than once a year, and (6) once a year.

**Figure 1:**
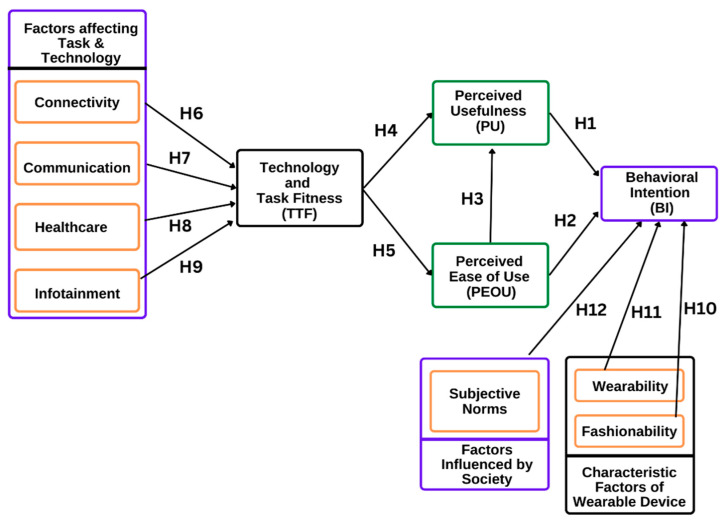
Proposed research model.

Correlation between technology-task fitness and connectivity, communication, healthcare, infotainment, perceived usefulness, and perceived ease of use was tested by calculation of the Pearson correlation coefficient with a 2-tailed significance level set at 5% (alpha < 0.05). Pearson correlation coefficient was calculated with a 2-tailed significance level set at 5% (alpha < 0.05) between behavioral intention and fashionability, s, Subjective norms, perceived usefulness, and perceived ease of use. An independent sample *t*-test was performed to compare mean scores of all factors in respondents who reported using wearable devices for healthcare versus those who reported not using them, to compare male versus female respondents, and between the less than 25 years age group and more than 25 years age group (*p* < 0.05). Figure 1 shows the proposed research model with the various factors considered to evaluate the influence of the usage or barriers of wearable device technology for healthcare.

## 3. Results

A total of 495 responses were obtained from the Google form questionnaire. General data for the participants are as follows: 65.5% of respondents were male and 34.5% were females; 50.3% were between 25–50 years of age; 51.3% reported being in the lower-income group (annual income less than Rs. 50,000 to Rs. 500,000); 62.2% of participants reported already using wearable devices for managing their health; 29.3% reported using wearable devices daily. Table 1 shows the demographic characteristics of the participants considered in the present study.

It was found that TTF moderately positively correlated with communication (*r* = 0.637) and infotainment (*r* = 0.598) and highly positively correlated with connectivity (*r* = 0.716) and health care (*r* = 0.780). Perceived usefulness (*r* = 0.792) and perceived ease of use (*r* = 0.800) were also found to be strongly correlated. Behavioral intention to use wearable devices was positively correlated to factors such as perceived usefulness, perceived ease of use, fashionability, wearability, and subjective norms. However, it was mildly correlated with fashionability (*r* = 0.472), moderately correlated with wearability (*r* = 0.642), subjective norms (*r* = 0.594), and perceived ease of use (*r* = 0.644), and highly correlated with perceived usefulness (*r* = 0.711). Table 2 shows the correlation values of the respective factors affecting technology and task fitness and behavioral intention in using wearable devices.

Table 3 shows that all factors have significantly higher mean scores among those participants who are already using wearable devices as compared to non-users (*p* < 0.001). There is no significant difference in mean scores of all the variables among the males and females except for behavioral intention to use wearable devices and perceived ease of use of devices. Males have significantly higher mean scores for behavioral intention (*p* = 0.034) and perceived ease of use (*p* = 0.009). There is no significant difference in mean scores of any of the variables among the two different age groups except for behavioral intention to use wearable devices and infotainment. The participants who are more than 25 years old have significantly higher mean scores for behavioral intention (*p* = 0.027) and infotainment (*p* = 0.032).

## 4. Discussion

In this survey, we analyzed whether socio-demographic and usage-determining factors correlated with self-reported use of wearable devices for healthcare in a subset of the Indian population, mainly medical and engineering professionals. This study provides empirical support for the hypothesis that factors, drawn from the TAM and TTF models along with additionally considered variables determining the use, are positively correlated with the self-reported use of wearable devices for healthcare.

### 4.1. Theoretical Models to Study the Acceptance of Technology among Users

Various theories of behavior have been formulated giving rise to different models predicting human behavior. A prominent and well-studied model is the technology acceptance model (TAM). This is based on a major theory of human behavior, the theory of reasoned action (TRA). Another common model is the task-technology fitness (TTF) model used to study the congruence of new information systems with task requirements.

The theory of reasoned action (TRA) states a person’s performance of a specified behavior is determined by their behavioral intention (BI) to perform it, which in turn is determined by the person’s attitude (A) and subjective norm (SN) concerning the behavior. According to the TAM model, two main factors influence the acceptance of new technology by users—perceived usefulness (PU) and perceived ease of use (PEOU). PU has been defined as “the degree to which a person believes that using a particular system would enhance their job performance”. PEOU has been defined as “the degree to which a person believes that using a particular system would be free from effort”. Both influence attitude (A) toward using the technology which in turn influences BI, which determines the actual usage [17]. PU and PEOU are independently correlated with a higher frequency of self-reported use of new information technology by users [17,18,19,20]. TAM was modified to incorporate SN from TRA, such that SN acted as external variables that affected PU and PEOU. The TAM model has been widely used to study the adoption of disparate projects in the field of information technology [20,21,22,23,24]. The TTF model proposes that user performance is improved if there is a congruence of the technology with the task at hand. It suggests that technology will be used and will improve user performance only if tool functionality fits task requirements [25].

TAM focuses on attitudes behind technology adoption, while TTF focuses on the operational aspects. Subsequent research has tried to integrate the TTF and TAM models to better explain technology acceptance and proposed that TTF factors influence PU and PEOU [26].

### 4.2. Variables Studied and Analyzed during Our Survey

A recent study by Chang et al. (2016) proposed a technology acceptance model for wearable healthcare devices based on the TAM–TTF model and defined TTF factors for wearable healthcare devices as connectivity, communication, healthcare, and infotainment. This study also proposed that external factors such as subjective norms and device factors of wearability and fashionability influence BI [27].

Drawing from the TAM and TTF models, we constructed an abbreviated questionnaire, similar to the previously used and validated construct by Chang et al., consisting of three-question items, each to assess BI, PU, PEOU, and TTF using the Likert scale. Different from the original TTF construct, this model assessed a subjective sense of task and technology fitness, rather than focused objective factors. Factors for TTF for wearable devices were used as defined by previous studies. Connectivity describes the interaction between devices using Bluetooth or wireless network technology. Communication refers to the function of wearable devices that allow users to communicate with other users, such as by making phone calls, text messaging, etc. Healthcare refers to how wearable devices assist the user in managing their health. This was assessed using a subjective three-question item construct. Infotainment refers to factors such as the displayed information about heart rate and distance statistics to guide improvement or seek enjoyment and motivation as users engage in health-promoting behavior. Fashionability refers to fashion factors related to the design of the wearable device. This has been seen as weakly significant or correlated in comparison with the use of wearable devices for healthcare management purposes. Wearability refers to design factors of the wearable device related to form and fit, ease of wearability, access to the device, etc. Wearability is strongly positively correlated with task-technology fitness. Subjective norms are social factors, such as what an individual who is important to the user thinks about the device. Evidence for subjective norms affecting the use of new technology has been seen to be more significant for female users in the older age group in the early stages of use, but these findings are more important in mandatory usage settings. In the case of voluntary use such as wearables, subjective norm falls to how it affects attitude and behavioral intention [28].

Using these models of human behavior as a base, we have tried to construct our theoretical model to predict the adoption of wearable devices for healthcare and gauge the response of a sub-section of the Indian population, as explained above. We have not elicited what type of wearable devices were used by our respondents, or how they used them for managing health. However, wearable fitness trackers with pedometers and accelerometers are the most common wearable devices used for healthcare found on the market worldwide, while in India, the market is largely dominated by “hearables”, smartwatches comprising the fastest growing device segment. This descriptive study largely applies to these devices [29,30].

### 4.3. Adoption of Wearable Healthcare Devices

Wearable devices and their specific use in healthcare management have been studied using various validated human behavior models, such as the TAM model, the successor UTAUT with protection motivation theory, and privacy calculus theory, which have recently evolved in the field to explain users’ privacy concerns [31,32]. These studies have analyzed various factors that influence the adoption, continued use, frequency of use, and discontinuation of wearable devices. A recent national survey in the USA, studying the reception of wearable device technology in the western world, estimated that close to 30% of adults are using wearable healthcare devices. This nationwide survey also correlated socio-economic, demographic, health, and technology, self-efficacy attributes to the actual use of wearable devices [33].

In the present study, we found that subjective measurements of task-technology fitness (TTF) are strongly positively correlated with device factors of connectivity and healthcare, and moderately positively correlated with communication and infotainment. This reflects the users’ perceptions that wearable devices are used mainly for healthcare, and for achieving health goals. The synchronization and connectivity of the wearable device to other devices are necessary for the ease of transfer of health data. Users/participants in the present study were not regularly using wearable healthcare devices for communication tasks such as making calls or messaging, as well as infotainment.

TTF measures were found to be strongly correlated with PU and PEOU. PU is strongly correlated with BI in previous studies using the TAM model on wearable devices and other technologies that are also in accordance with our study [20]. PEOU is less strongly correlated, which may be due to the moderating influence of PU on PEOU, which has been well described in the literature [17,25]. Wearability and subjective norm were moderately positively correlated with BI, and fashionability is less correlated with BI. This is similar to previous study observations, indicating that the wearability of the device, along with the perception of other people about wearable devices, significantly influences Behavioral intention to use them, though fashionability does not significantly affect it [27]. This shows the practicality aspect of users’ intention that wearable devices are preferred for health care management rather than fashion sense.

All factors determining use were positively correlated with reported use, as respondents who were using wearable healthcare devices had higher mean scores than non-users. This gives an insight, that current users were happy with their product and hence were more motivated to use it. Males had a higher BI and PEOU than females. This follows a similar trend observed in the previous studies using TAM, and UTAUT models, which found females to have more difficulty learning how to operate new information technology and have lower scores of PEOU or higher scores of perceived difficulties [25,26,27]. The authors discussed the social context behind this, and they had hoped that this gap would reduce in the internet age. This may apply to India, but the Indian demographic may be more susceptible to lingering effects of gender disparity, opportunity, and exposure. Differences in other factors were non-significant. While wearable devices are more common among females, there is a mutually constitutive relationship between gender and technology, which in turn is adapted by technological transformations. It also means that societies with better gender equality also have a better digital economy.

Our study findings show an interesting trend where adults aged more than 25 years showed higher BI and infotainment. This may reflect changing perceptions around wearables for personal health management and fitness among older adults in India. This gives further reason for supporting the adoption of wearables as a cost-effective means of monitoring physical activity and maintaining general health.

The original TAM model pilot study used a 10-question item construct to measure PU and PEOU. This model was abbreviated in the present study to three questions per domain. The original UTAUT study used measures of performance expectancy, effort expectancy, and social influence that act on behavioral intention, which determines usage. Performance expectancy is a similar construct to PU, and effort expectancy to PEOU in TAM. Social norms in TAM2 have been seen to be similar to social influence in UTAUT. The study included three-question items similar to those used in the final UTAUT question construct, which was formulated to ensure the highest object loading and degrees of freedom according to psychometric theories, which may compromise content validity due to insufficient representation of the content [26,27].

Privacy remains a major point of concern for consumers interested in wearable healthcare devices. In particular, the release of personal information and data for analysis carries the risk of dissemination, leak, and unauthorized use [34,35,36]. A distinct challenge that arises in India is the heterogeneity in the availability and quality of devices. Consumer devices are not subjected to regulatory frameworks and rigorous testing that medical devices typically undergo, which poses concerns about the validity of device recordings and data security concerns. The worldwide wearable devices market ballooned in 2014 and has since resulted in a plethora of device types with different sensors, software, and design that have entered the Indian market as well. This adds additional factors that contribute to the acceptance and usage of devices, including the type and accuracy of sensors, the complexity of the user interface, and brand value perception. Only a few brands have been used and validated in formal research studies. Researchers have developed a comprehensive device evaluation tool that may be used to guide future regulatory policies [37]. Another challenge is the high attrition rate and fall in the usage of wearable devices over time. A 2016 survey found that 30% of users of a popular brand name fitness tracker discontinue use within 6 months [38]. A 2019 study found that 20% of users abandoned their devices, with the most common reasons cited to be related to data literacy, or device comfort [39]. Age and limited technology literacy, with issues related to perceived measurement inaccuracy, have been seen to be major factors for device abandonment, and pose a significant challenge to behavioral change and long-term healthcare management goals [40,41]. Behavioral change techniques (BCT) such as just-in-time adaptive interventions, for example, motivational mobile messages accompanying device notifications and gamification, have been seen to be effective at increasing physical activity and may help solidify behavioral changes [42,43].

### 4.4. Limitations

The survey was primarily circulated among professionals interested in technology and personal healthcare technology, and this may be a source of sampling bias. Since we only examined a narrow subset of the population in the context of India, the generalizability and interpretation cannot be extrapolated to other contexts as in different countries or different social backgrounds. Although we compared differences across gender and age groups, we did not look into and compare differences across income groups. Moreover, all questionnaire responses are self-reported, and reflect subjective perceptions about factors and therefore may be subject to interpretation bias by participants. This is a questionnaire survey, hence cannot elicit development, use, or loss to attrition of wearable healthcare devices use, which has been elucidated in multiple previous studies [23,24]. Hence, conducting longitudinal studies will better address the issue of factors determining long-term use. Our model is constructed based on the TAM and TTF models. Although aspects of privacy calculus theory such as hedonic motivation, performance expectancy, etc. can be equated to similar measures used in TAM and TTF, it does not include the privacy calculus model, which also addresses concerns regarding personal health data security and privacy.

## 5. Conclusions

The use of wearable healthcare device usage has skyrocketed in India over the past few years. This is important for the medicine and healthcare industry because wearable devices play an important role in monitoring and preventing chronic diseases to a certain level. In a developing country such as India, diseases and hospitalization make a major impact on the financial status of the family, in turn affecting their quality of life. The present study helps in filling the significant research gap of studies looking at the adoption and acceptance of wearable devices in the context of a low-middle-income country.

## Figures and Tables

**Table 1:** Demographic characteristics of study participants.

Variables		Number of Participants	Percentage (%)
Gender	Male	324	65.5
	Female	171	34.5
Age (in Years)	Less than 18	22	4.4
	18–25	186	37.6
	25–50	249	50.3
	More than 50	38	7.7
Income	Lower (Less than 50,000–500,000)	254	51.3
	Middle (500,000–2,500,000)	172	34.7
	Upper Middle (2,500,000–5,000,000)	44	8.9
	Elite (Greater than 5,000,000)	25	5.1
Are you currently using wearable devices for your healthcare?	No	187	37.8
	Yes	308	62.2
How frequently do you use wearable healthcare devices?	Once a year	145	29.3
	More than once a year	33	6.7
	Once a month	62	12.5
	Once or twice in 3 months	39	7.9
	Once or twice a week	71	14.3
	Daily	145	29.3

**Table 2:** Factors affecting technology and task fitness (TTF) and Behavioral Intention to use Wearable Devices [N = 495].

Variable	Pearson Correlation	Sig. (2-Tailed)
**Technology and Task Fitness**		
Connectivity	0.716 **	<0.001
Communication	0.637 **	<0.001
Health Care	0.780 **	<0.001
Infotainment	0.598 **	<0.001
Perceived Usefulness	0.792 **	<0.001
Perceived Ease of Use	0.800 **	<0.001
**Behavioral Intention**		
Fashionability	472 **	<0.001
Wearability	0.642 **	<0.001
Subjective Norms	0.594 **	<0.001
Perceived Usefulness	0.711 **	<0.001
Perceived Ease of Use	0.644 **	<0.001

** Correlation is significant at the 0.01 level (2-tailed).

**Table 3:** Mean scores of different factors affecting the use of wearable devices across already usage of wearable devices, gender, and age.

Variables	Already Using Wearable Devices for Healthcare		*p*-Value	Gender		*p*-Value	Age		*p*-Value
	Yes (N = 308)	No (N = 187)		Male (N = 324)	Female (N = 171)		Less than 25 Years (N = 207)	More than 25 Years (N = 287)	
	Mean (SD)	Mean (SD)		Mean (SD)	Mean (SD)		Mean (SD)	Mean (SD)	
Behavioral Intention	12.47 (2.4)	10.88 (3.1)	<0.001 *	12.06 (2.6)	11.50 (3.1)	0.034 *	11.54 (2.6)	12.11 (2.9)	0.027 *
Perceived Usefulness	13.09 (2.3)	10.43 (3.4)	<0.001 *	12.20 (2.9)	11.86 (3.4)	0.24	11.83 (2.9)	12.26 (3.1)	0.122
Perceived Ease of Use	13.36 (2.2)	11.17 (3.7)	<0.001 *	12.79 (2.8)	12.04 (3.4)	0.009 *	12.51 (3.0)	12.54 (3.0)	0.910
Technology and Task Fitness	13.17 (2.0)	11.02 (2.9)	<0.001 *	12.49 (2.5)	12.09 (2.9)	0.118	12.09 (2.5)	12.55 (2.7)	0.06
Connectivity	13.06 (2.4)	10.98 (3.4)	<0.001 *	12.40 (2.9)	12.02 (3.2)	0.185	12.21 (2.9)	12.32 (3.1)	0.691
Communication	12.23 (3.2)	10.49 (3.8)	<0.001 *	11.67 (3.5)	11.39 (3.6)	0.413	11.25 (3.5)	11.89 (3.6)	0.08
Health Care	13.39 (1.9)	11.65 (3.1)	<0.001 *	12.84 (2.5)	12.52 (2.8)	0.196	12.67 (2.5)	12.77 (2.6)	0.684
Infotainment	11.68 (3.4)	10.35 (3.8)	<0.001 *	11.15 (3.6)	11.23 (3.6)	0.794	10.77 (3.6)	11.47 (3.5)	0.032 *
Fashionability	11.85 (3.2)	10.87 (3.5)	0.002 *	11.62 (3.2)	11.22 (3.7)	0.219	11.74 (3.1)	11.30 (3.5)	0.156
Wearability	12.52 (2.3)	11.26 (2.8)	<0.001 *	12.14 (2.5)	11.86 (2.8)	0.255	12.16 (2.4)	11.96 (2.8)	0.410
Subjective Norms	11.37 (3.6)	10.06 (3.7)	<0.001 *	10.92 (3.8)	10.79 (3.7)	0.714	10.58 (3.8)	11.09 (3.7)	0.139

Independent Sample *t*-test, * *p*-value < 0.05 is considered statistically significant.

## Data Availability

All data and material collected are presented in the manuscript. Clarification on any matter can be made through the corresponding author.

## Supplementary Materials

The following supporting information can be downloaded at: https://www.mdpi.com/article/10.3390/jcm11237019/s1. Use of Wearable Health Care Devices by Adults in Managing Personal Health—A Questionnaire.

## References

[1] Lu L., Zhang J., Xie Y., Gao F., Xu S., Wu X., Ye Z. (2020). Wearable Health Devices in Health Care: Narrative Systematic Review. JMIR mHealth uHealth.

[2] Mills M. (2011). Hearing aids and the history of electronics miniaturization. IEEE Ann. Hist. Comput..

[3] Oswald A. (2014). At the Heart of the Invention: The development of the Holter Monitor.

[4] Ioannou K., Ignaszewski M., Macdonald I. (2014). Ambulatory electrocardiography: The contribution of Norman Jefferis Holter. BC Med. J..

[5] Kvedar J., Coye M.J., Everett W. (2014). Connected Health: A Review of Technologies and Strategies to Improve Patient Care with Telemedicine and Telehealth. Health Aff..

[6] Ray P.P., Dash D., De D. (2017). A Systematic Review of Wearable Systems for Cancer Detection: Current State and Challenges. J. Med. Syst..

[7] Li X., Dunn J., Salins D., Zhou G., Zhou W., Rose S.M.S.-F., Perelman D., Colbert E., Runge R., Rego S. (2017). Digital Health: Tracking Physiomes and Activity Using Wearable Biosensors Reveals Useful Health-Related Information. PLoS Biol..

[8] Lapointe J., Bécotte-Boutin H.-S., Gagnon S., Levasseur S., Labranche P., D’Auteuil M., Abdellatif M., Li M.-J., Vallée R. (2021). Smartphone Screen Integrated Optical Breathalyzer. Sensors.

[9] Pyrkov T.V., Slipensky K., Barg M., Kondrashin A., Zhurov B., Zenin A., Pyatnitskiy M., Menshikov L., Markov S., Fedichev P.O. (2018). Extracting biological age from biomedical data via deep learning: Too much of a good thing?. Sci. Rep..

[10] Moon J.H., Kang M.-K., Choi C.-E., Min J., Lee H.-Y., Lim S. (2020). Validation of a wearable cuff-less wristwatch-type blood pressure monitoring device. Sci. Rep..

[11] Franssen W., Franssen G.H.L.M., Spaas J., Solmi F., Eijnde B.O. (2020). Can consumer wearable activity tracker-based interventions improve physical activity and cardiometabolic health in patients with chronic diseases? A systematic review and meta-analysis of randomised controlled trials. Int. J. Behav. Nutr. Phys. Act..

[12] Yen H.-Y. (2021). Smart wearable devices as a psychological intervention for healthy lifestyle and quality of life: A randomized controlled trial. Qual. Life Res..

[13] Saini G., Budhwar V., Choudhary M. (2022). Review on people’s trust on home use medical devices during COVID-19 pandemic in India. Health Technol..

[14] Koo S.H. (2017). Consumer Differences in the United States and India on Wearable Trackers. Fam. Consum. Sci. Res. J..

[15] Pandey S., Chawla D., Puri S., Jeong L.S. (2021). Acceptance of wearable fitness devices in developing countries: Exploring the country and gender-specific differences. J. Asia Bus. Stud..

[16] Devine J.K., Schwartz L.P., Choynowski J., Hursh S.R. (2022). Expert Demand for Consumer Sleep Technology Features and Wearable Devices: A Case Study. IoT.

[17] Jeong J.-Y., Roh T.-W. (2017). The Intention of Using Wearable Devices: Based on Modified Technology Acceptance Model. J. Digit. Converg..

[18] Adams D.A., Nelson R.R., Todd P.A. (1992). Perceived Usefulness, Ease of Use, and Usage of Information Technology: A Replication. MIS Q..

[19] Szajna B. (1994). Software Evaluation and Choice: Predictive Validation of the Technology Acceptance Instrument. MIS Q..

[20] Rahimi B., Nadri H., Afshar H.L., Timpka T. (2018). A Systematic Review of the Technology Acceptance Model in Health Informatics. Appl. Clin. Inform..

[21] Verdru J., Van Paesschen W. (2020). Wearable seizure detection devices in refractory epilepsy. Acta Neurol. Belg..

[22] Nelson B.W., Allen N.B. (2019). Accuracy of Consumer Wearable Heart Rate Measurement During an Ecologically Valid 24-Hour Period: Intraindividual Validation Study. JMIR mHealth uHealth.

[23] Ocagli H., Lorenzoni G., Lanera C., Schiavo A., D’Angelo L., Di Liberti A., Besola L., Cibin G., Martinato M., Azzolina D. (2021). Monitoring Patients Reported Outcomes after Valve Replacement Using Wearable Devices: Insights on Feasibility and Capability Study: Feasibility Results. Int. J. Environ. Res. Public Health.

[24] Rodriguez-León C., Villalonga C., Munoz-Torres M., Ruiz J.R., Banos O. (2021). Mobile and wearable technology for the monitoring of diabetes-related parameters: Systematic review. JMIR mHealth and uHealth.

[25] Goodhue D.L., Thompson R.L. (1995). Task-Technology Fit and Individual Performance. MIS Q..

[26] Dishaw M.T., Strong D.M. (1999). Extending the technology acceptance model with task–technology fit constructs. Inf. Manag..

[27] Chang H.S., Lee S.C., Ji Y.G. (2016). Wearable device adoption model with TAM and TTF. Int. J. Mob. Commun..

[28] Venkatesh V., Morris M.G., Davis G.B., Davis F.D. (2003). User acceptance of information technology: Toward a unified view. MIS Q..

[29] Bove L.A. (2019). Increasing Patient Engagement Through the Use of Wearable Technology. J. Nurse Pract..

[30] Bhargava S., Gupta P. (2022). Boat: The Indian startup scripts a revolutionizing growth strategy. Emerald Emerg. Mark. Case Stud..

[31] Gao Y., Li H., Luo Y. (2015). An empirical study of wearable technology acceptance in healthcare. Ind. Manag. Data Syst..

[32] Al-Maroof R.S., Alhumaid K., Alhamad A.Q., Aburayya A., Salloum S. (2021). User Acceptance of Smart Watch for Medical Purposes: An Empirical Study. Future Internet.

[33] Chandrasekaran R., Katthula V., Moustakas E. (2020). Patterns of Use and Key Predictors for the Use of Wearable Health Care Devices by US Adults: Insights from a National Survey. J. Med. Internet Res..

[34] Cheatham S.W., Stull K.R., Fantigrassi M., Motel I. (2018). The efficacy of wearable activity tracking technology as part of a weight loss program: A systematic review. J. Sports Med. Phys. Fit..

[35] Fawcett E., Van Velthoven M.H., Meinert E. (2020). Long-Term Weight Management Using Wearable Technology in Overweight and Obese Adults: Systematic Review. JMIR mHealth uHealth.

[36] Maddison R., Cartledge S., Rogerson M., Goedhart N.S., Singh T.R., Neil C., Phung D., Ball K. (2019). Usefulness of Wearable Cameras as a Tool to Enhance Chronic Disease Self-Management: Scoping Review. JMIR mHealth uHealth.

[37] Bayoumy K., Gaber M., Elshafeey A., Mhaimeed O., Dineen E.H., Marvel F.A., Martin S.S., Muse E.D., Turakhia M.P., Tarakji K.G. (2021). Smart wearable devices in cardiovascular care: Where we are and how to move forward. Nat. Rev. Cardiol..

[38] Gartner Survey Shows Wearable Devices Need to Be More Useful [Internet]. https://www.gartner.com/en/newsroom/press-releases/2016-12-07-gartner-survey-shows-wearable-devices-need-to-be-more-useful.

[39] Jarusriboonchai P., Häkkilä J. Customisable wearables: Exploring the design space of wearable technology. Proceedings of the 18th International Conference on Mobile and Ubiquitous Multimedia 2019.

[40] Steinert A., Haesner M., Steinhagen-Thiessen E. (2018). Activity-tracking devices for older adults: Comparison and preferences. Univers. Access Inf. Soc..

[41] Kononova A., Li L., Kamp K., Bowen M., Rikard R.V., Cotten S., Peng W. (2019). The Use of Wearable Activity Trackers among Older Adults: Focus Group Study of Tracker Perceptions, Motivators, and Barriers in the Maintenance Stage of Behavior Change. JMIR mHealth uHealth.

[42] Martin S.S., Feldman D.I., Blumenthal R.S., Jones S.R., Post W.S., McKibben R.A., Michos E.D., Ndumele C.E., Ratchford E.V., Coresh J. (2015). mActive: A randomized clinical trial of an automated mHealth intervention for physical activity promotion. J. Am. Heart Assoc..

[43] Patel M.S., Benjamin E.J., Volpp K.G., Fox C.S., Small D.S., Massaro J.M., Lee J.J., Hilbert V., Valentino M., Taylor D.H. (2017). Effect of a game-based intervention designed to enhance social incentives to increase physical activity among families: The BE FIT randomized clinical trial. JAMA Intern. Med..

