# Supplementary material for: Factors Affecting the Usage of Wearable Device Technology for Healthcare among Indian Adults: A Cross-Sectional Study
Source: J Clin Med. 2022 Nov 28;11(23):7019. doi: 10.3390/jcm11237019 (PMC9740494; doi:10.3390/jcm11237019)
Supplement: Supplementary file 1 [file jcm-11-07019-s001.zip › jcm-2013735-supplementary.pdf]

Use of Wearable Health Care Devices by Adults

in Managing Personal Health - A Questionnaire

Personal Data (Optional)

Name:

About this Research

Health is a state of mind or body free from illness and injury. In this connection this research is aiming at the identification of Factors affecting the usage of wearable device technology for healthcare which significantly influence an adult's state of well-being. The study protocols approved by the Institutional Ethical committee (ethical approval number FMIEC- 94/2022).

Confidentiality Statement

The data in the questionnaire shall be used solely for academic research purposes. No personal details of the respondent shall be used anywhere.

Questions

If you require assistance in completing this questionnaire or have any questions regarding the survey, please contact;

Socio-economic Details

Gender ☐ Male ☐ Female

Age ☐ <18 years ☐ 18-25 years ☐ 25-50 years ☐ More than 50 years

Qualification ☐ 10+2 ☐ Graduate ☐ Post-graduate ☐ Diploma

Already using Healthcare Wearable devices? Yes ☐ No ☐

How frequently do you use wearable healthcare devices?

Daily ☐

Once or twice a week ☐

Once or twice in 3 months ☐

Once a month ☐

More than once a year ☐

Once a year ☐

Income Group (Rs.) ☐ Lower (Less than INR 50,000 – 5,00,000)

☐ Middle (INR 5,00,000 - 25,00,000)

☐ Upper Middle (INR 25,00,000 - 50,00,000)

☐ Elite (Greater than INR 50,00,000)

Place Tick mark (√) on ONE response for each item based on your experiences with mobile phones.

**5- Strongly Agree**	**1– Strongly Disagree**	**5**	**4**	**3**	**2**	**1**
**1. Behavioural Intention (BI)**						
BI1	I try the usage of wearable devices whenever I get a chance.					
BI2	I am likely to use wearable devices later in life					
BI3	I intend to use wearable devices only for specific or chronic illness.					
**2. Perceived Usefulness (PU)**						
PU1	Wearable health care devices help me in my day-to-day activities					
PU2	Using wearable devices makes my daily task performances more efficient					
PU3	Wearable devices are beneficial to me					
**3. Perceived Ease of Use (PEOU)**						
PEOU1	The wearable device that I own can be handled easily					
PEOU2	It is easy to learn how to use the wearable device that I own					
PEOU3	The wearable device that I own can be easily accessed at any time					

**4. Technology and Task Fitness (TTF)**						
TTF1	I am happy with the functionalities of wearable devices.					
TTF2	In helping me to perform the assigned task, the functionalities of the wearable devices are always adequate					
TTF3	I want my healthcare wearables to have some more engaging functions.					
**5. Connectivity**						
CON1	It is useful to connect wearable devices with other gadgets					
CON2	It is useful if the applications of my smart devices are synchronized					
CON3	It is useful to use wearable devices with other smart devices					
**6. Communication**						
COM1	I need to check messages, e-mails, or phone calls using wearable					
COM2	I intend to use the wearable device to interact with my friends, family, and colleagues					
COM3	Messaging through wearable devices enables me to respond to my friends, family, and colleagues					
**7. Healthcare**						
HEA1	It is useful for me to use wearable devices for health management and completing my goals					
HEA2	I am interested in managing my health					
HEA3	It is useful for me to use healthcare applications through wearable devices					
**8. Infotainment**						

GRA1	It is useful for me to use wearable devices for checking SNS (Simple Notification System) and surf the web					
GRA2	It is useful for me to use entertainment applications through wearable					
GRA3	I want the usage of gadgets to search and play game applications					
**9. Fashionability**						
FAS1	I think wearable devices are fashion items					
FAS2	The outward show of gadget is of importance to me					
FSA3	The design and aesthetics of wearable devices are important to me					
**10. Wearability**						
WEA1	The fit and overall comfort of wearable devices are important for me					
WEA2	I am not ready to use gadgets that feel uncomfortable					
WEA3	I am ready to use wearable healthcare gadgets regardless of how they feel Subjective					
**11. Subjective Norm (SN)**						
SN1	People whom I care about think usage of wearable devices is good.					
SN2	Usage of wearable gadgets is recommended by my friends and family.					
SN3	Usage of wearable devices is recommended by my friends					
